# Supplementary material for: Multilocus Phylogeography of the Tuber mesentericum Complex Unearths Three Highly Divergent Cryptic Species
Source: J Fungi (Basel). 2021 Dec 17;7(12):1090. doi: 10.3390/jof7121090 (PMC8704588; doi:10.3390/jof7121090)
Supplement: Supplementary file 1 [file jof-07-01090-s001.zip › Supplementary figures with captions.pdf]

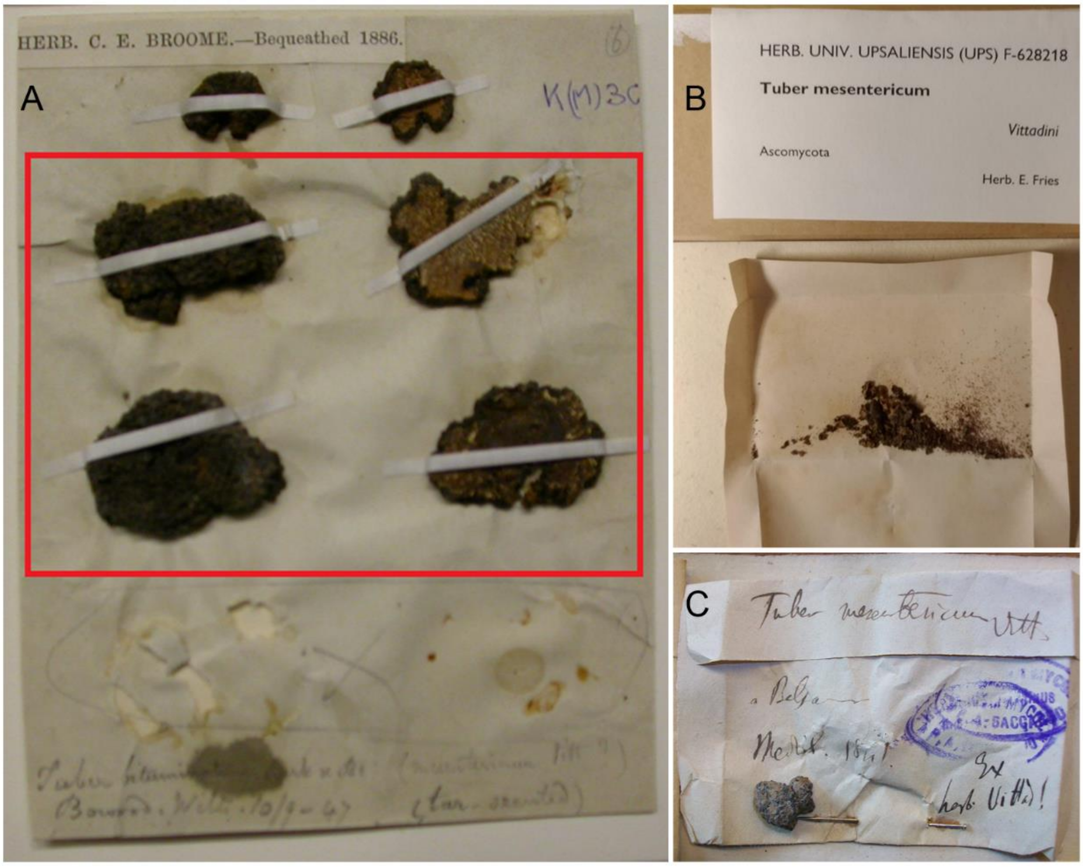

Figure S1. Historical voucher specimens from kew (**A**), Uppsala (**B**) and Padua (**C**) herbaria.

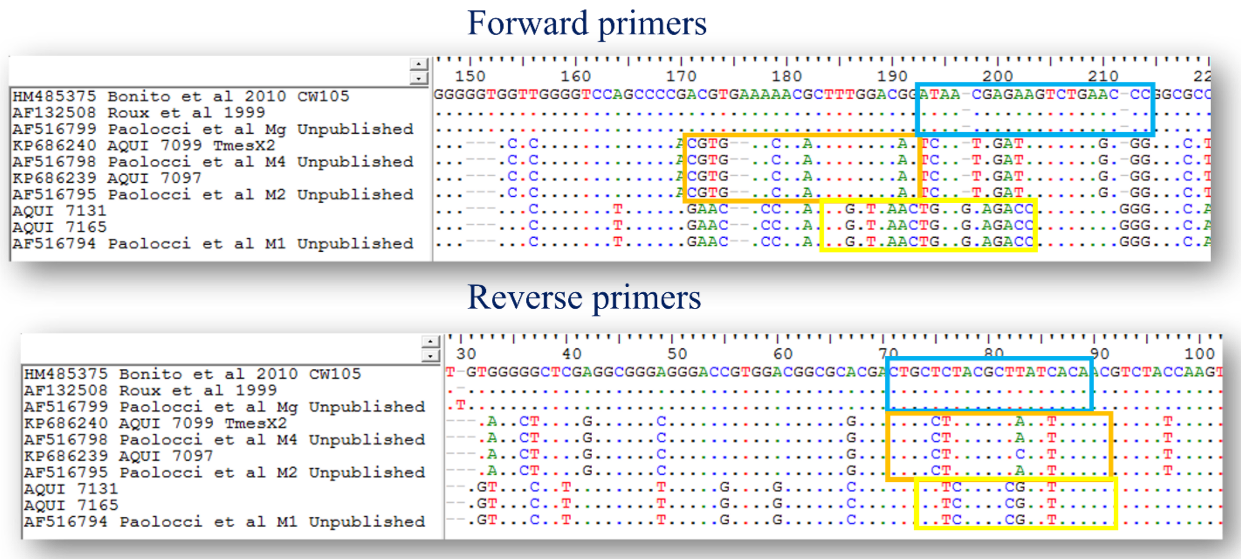

Figure S2. Localization of the three *T. mesentericum* clade-specific primer pairs on the ITS region alignment

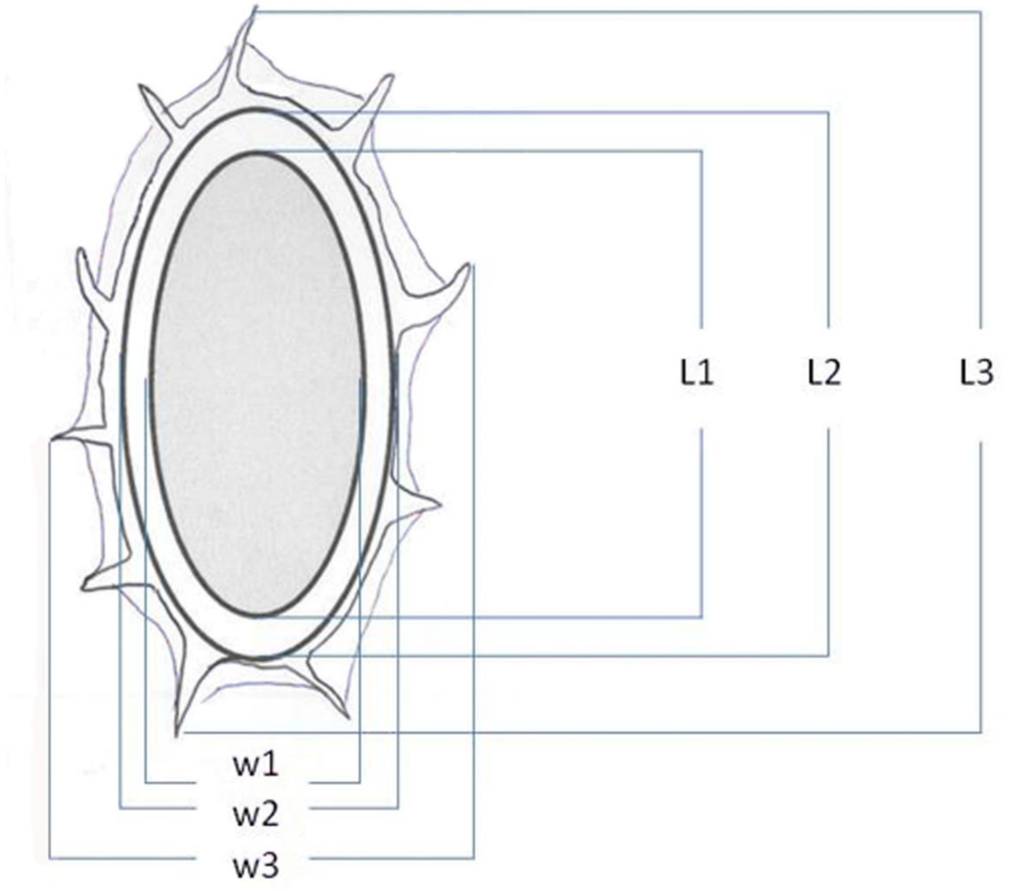

Figure S3. Graphic representation of spore parameters measured in this study.

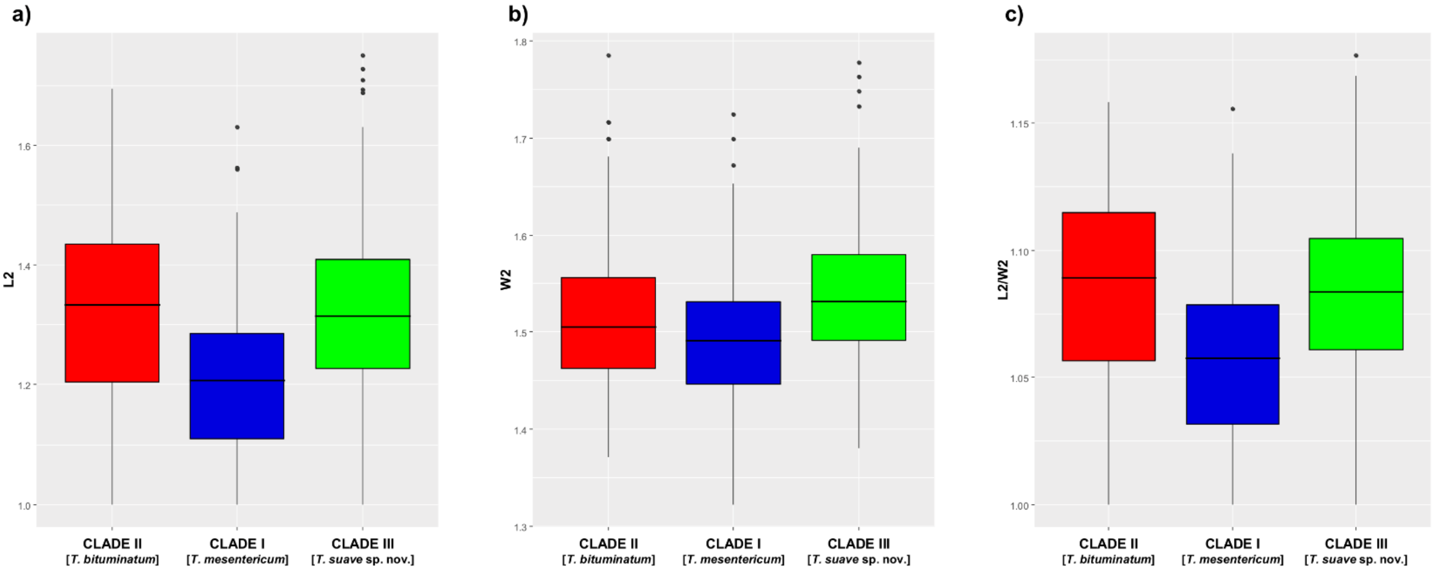

Figure S4. Boxplots of sporal dimensions (**A**, L2-lenght; **B**, W2-width; **C,** L2/W2 ratio) in the three clades.
