# Supplementary material for: Multilocus Phylogeography of the Tuber mesentericum Complex Unearths Three Highly Divergent Cryptic Species
Source: J Fungi (Basel). 2021 Dec 17;7(12):1090. doi: 10.3390/jof7121090 (PMC8704588; doi:10.3390/jof7121090)
Supplement: Supplementary file 1 [file jof-07-01090-s001.zip › Table S2.docx]

**Table S2.** Results of one-way ANOVA with repeated measure for differences in spore size (L2, W2 and L2/W2; log-transformed data) among the three cryptic lineages.

| **Parameters** |  | **Sum of Squares** | **df** | **F** | ***p*** |
| --- | --- | --- | --- | --- | --- |
| L2 | Species | 1.424 | 2 | 3.86 | 0.051 |
|  | Residuals (between) | 2.216 | 12 |  |  |
|  | Residuals (within) | 8.512 | 478 |  |  |
| W2 | Species | 0.208 | 2 | 2.88 | 0.095 |
|  | Residuals (between) | 0.434 | 12 |  |  |
|  | Residuals (within) | 2.233 | 478 |  |  |
| L2/W2 | Species | 0.084 | 2 | 3.53 | 0.062 |
|  | Residuals (between) | 0.143 | 12 |  |  |
|  | Residuals (within) | 0.498 | 478 |  |  |
