# Supplementary material for: Multilocus Phylogeography of the Tuber mesentericum Complex Unearths Three Highly Divergent Cryptic Species
Source: J Fungi (Basel). 2021 Dec 17;7(12):1090. doi: 10.3390/jof7121090 (PMC8704588; doi:10.3390/jof7121090)
Supplement: Supplementary file 1 [file jof-07-01090-s001.zip › Table S2.pdf]

Table S2. Results of one-way ANOVA with repeated measure for differences in spore size (L2, W2 and L2/W2; log-transformed data) among the three cryptic lineages.

Parameters		Sum of Squares	df	F	*p*
L2	Species	1.424	2	3.86	0.051
	Residuals (between)	2.216	12		
	Residuals (within)	8.512	478		
W2	Species	0.208	2	2.88	0.095
	Residuals (between)	0.434	12		
	Residuals (within)	2.233	478		
L2/W2	Species	0.084	2	3.53	0.062
	Residuals (between)	0.143	12		
	Residuals (within)	0.498	478		
