# Supplementary figures and images for: Multilocus Phylogeography of the Tuber mesentericum Complex Unearths Three Highly Divergent Cryptic Species
Source: J Fungi (Basel). 2021 Dec 17;7(12):1090. doi: 10.3390/jof7121090 (PMC8704588; doi:10.3390/jof7121090)

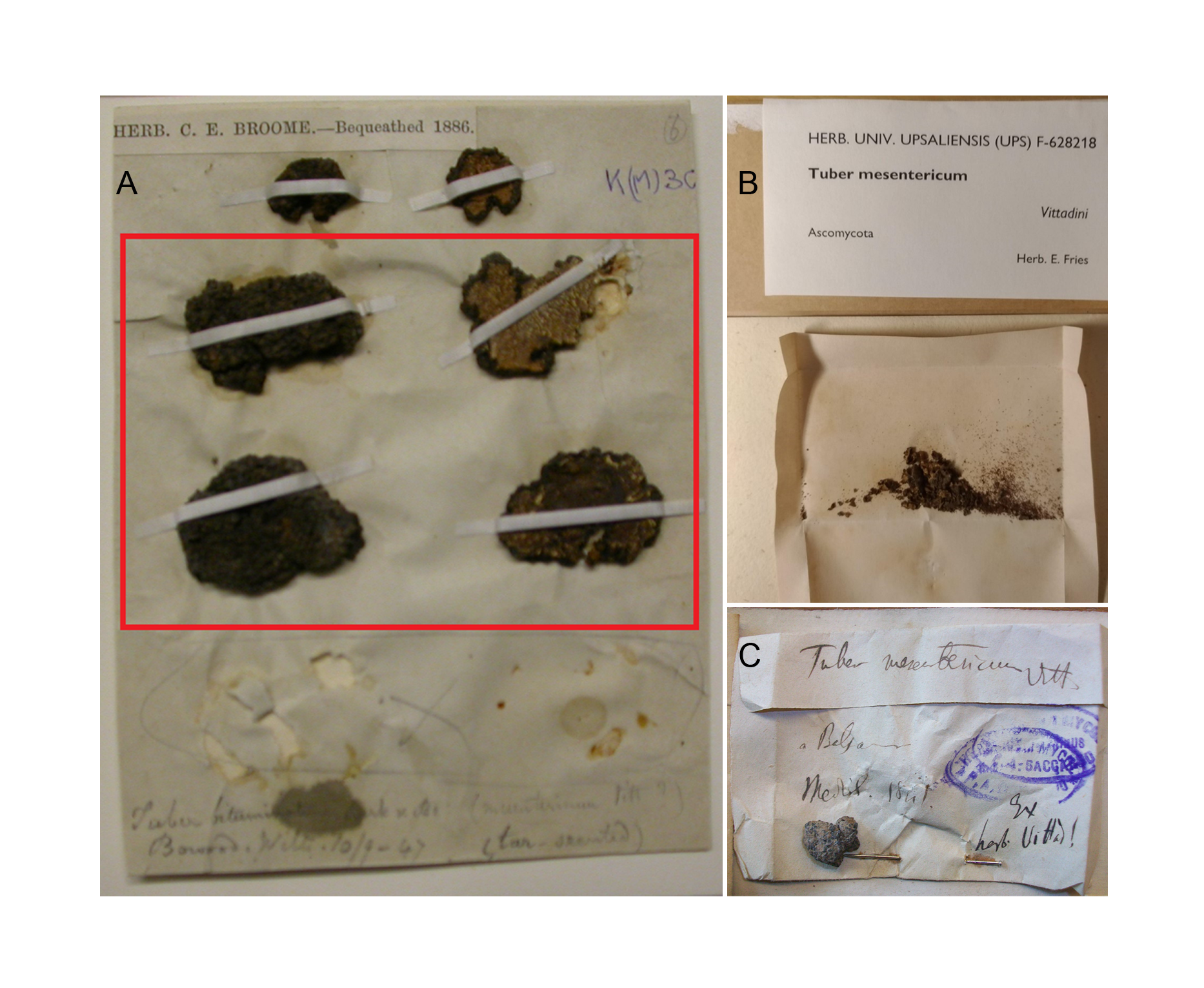

Supplement: Supplementary file 1 [file jof-07-01090-s001.zip › Figure S1.tif]

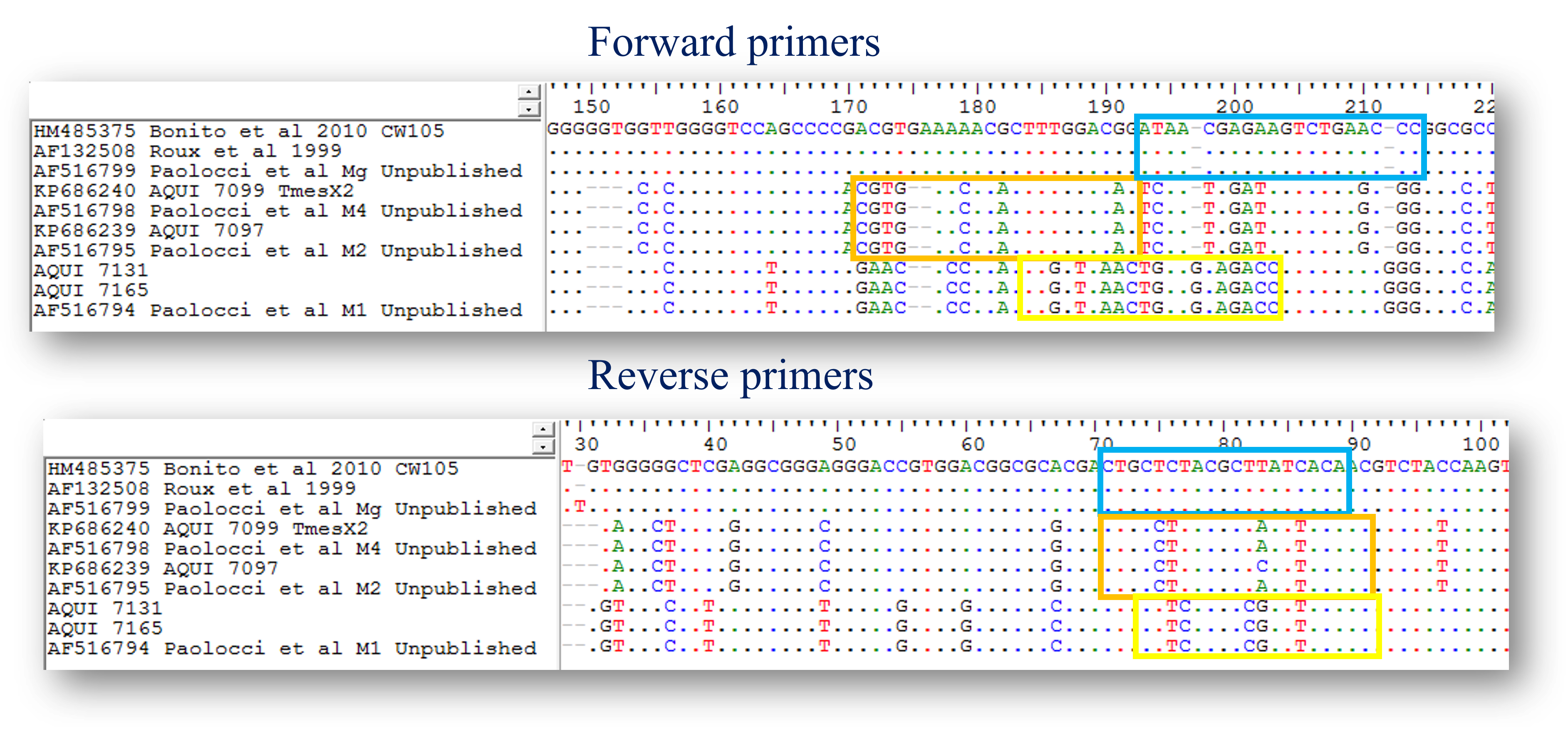

Supplement: Supplementary file 1 [file jof-07-01090-s001.zip › Figure S2.tif]

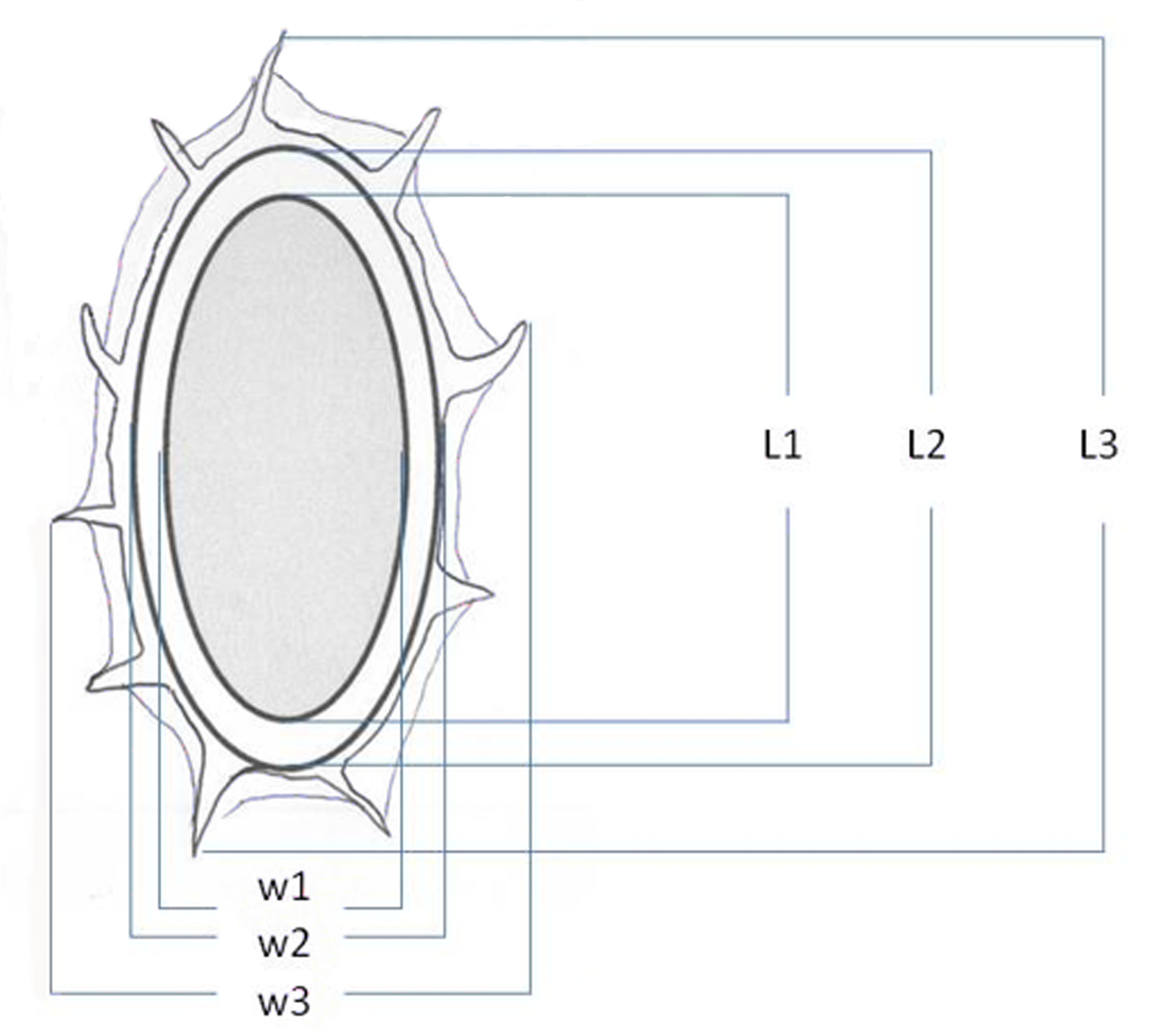

Supplement: Supplementary file 1 [file jof-07-01090-s001.zip › Figure S3.tif]

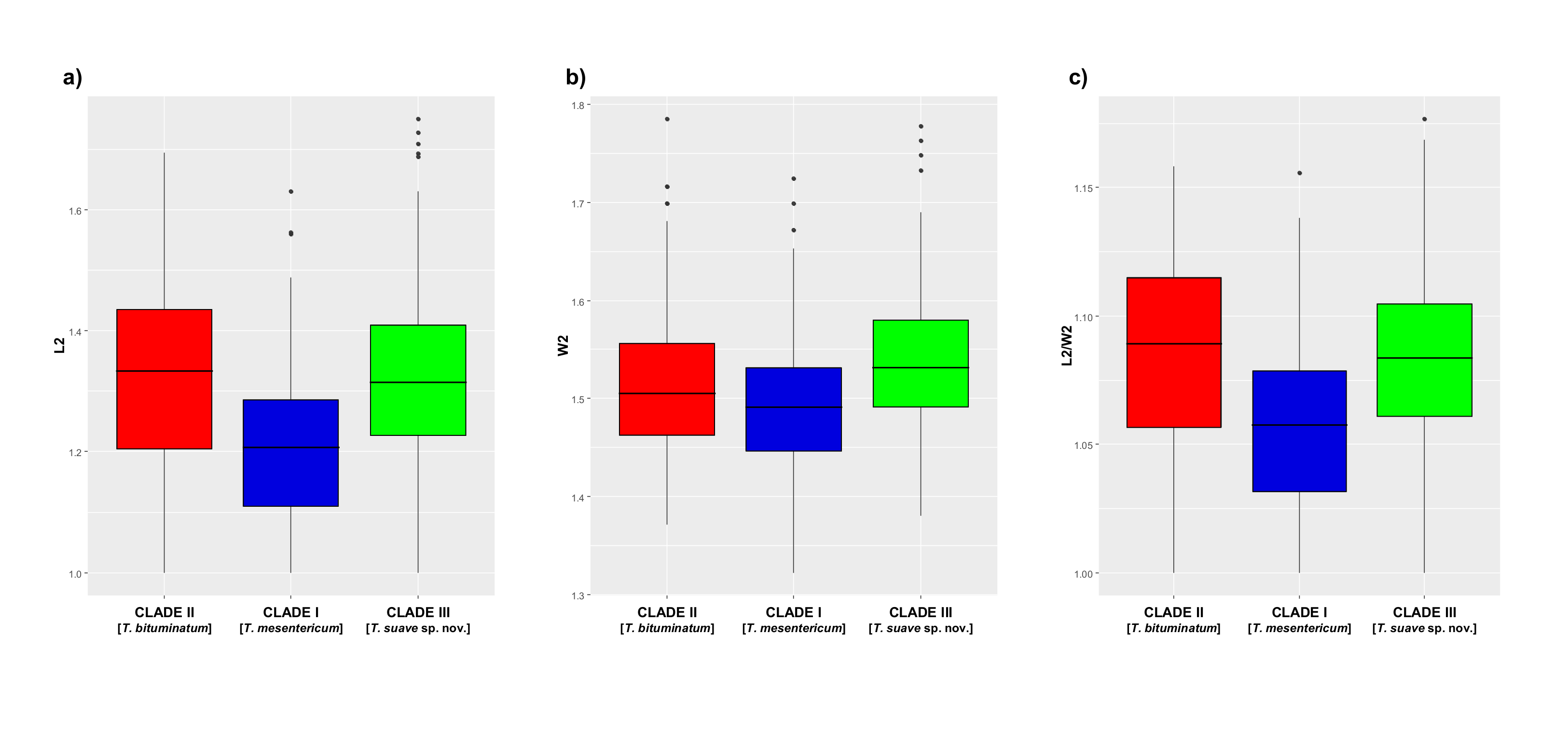

Supplement: Supplementary file 1 [file jof-07-01090-s001.zip › Figure S4.tif]
